# A machine learning approach for detecting WPA3 downgrade attacks in next-generation Wi-Fi systems

**DOI:** 10.1371/journal.pone.0331443

**Published:** 2025-09-02

**Authors:** Aya Tareef, Yazan M. Allawi, Anas A. Alkasasbeh, Ahmad Abadleh, Wasan Alamro, Mansoor Alghamdi, Aymen I. Zreikat, Hunseok Kang

**Affiliations:** 1 CS Dept., Mutah University, Jordan; 2 Department of Electrical Engineering, College of Engineering, Princess Nourah bint Abdulrahman University, P.O. Box 84428, Riyadh 11671, Saudi Arabia; 3 CS Dept., Applied College, University of Tabuk, Tabuk, Saudi Arabia; 4 Department of Computer and Communication Engineering, Faculty of Engineering, Al-Ahliyya Amman University, Amman, Jordan; 5 College of Engineering and Technology, American University of the Middle East, Kuwait; National University of Sciences and Technology, UNITED KINGDOM OF GREAT BRITAIN AND NORTHERN IRELAND

## Abstract

This paper presents a hybrid adaptive approach based on machine learning (ML) for classifying incoming traffic, feature selection and thresholding, aimed at enhancing downgrade attack detection in Wi-Fi Protected Access 3 (WPA3) networks. The fast proliferation of WPA3 is regarded critical for securing modern Wi-Fi systems, which have become integral to 5G and Beyond (5G&B) Radio Access Networks (RAN) architecture. However, the wireless communication channel remains inherently susceptible to downgrade attacks, where adversaries intentionally cause networks to revert from WPA3 to WPA2, with the malicious intent of exploiting known security flaws. Traditional Intrusion Detection Systems (IDS), which rely on fixed-threshold statistical methods, often fail to adapt to changing network environments and new, sophisticated attack strategies. To address this limitation, we introduce a novel ML-based Feature Selection and Thresholding for Downgrade Attacks Detection (MFST-DAD) approach, which comprises three stages: traffic data preprocessing, baseline adaptive feature selection, and real-time detection and prevention using ML algorithms. Experimental results on a specially generated dataset demonstrate that the proposed approach detects downgrade attacks in WPA3 networks, achieving 99.8% accuracy with a Naive Bayes classifier in both WPA3 personal and enterprise transition modes. These findings confirm the effectiveness of our proposed approach in securing next-generation Wi-Fi systems.

## 1 Introduction

The rapid advancement of wireless communication technologies has significantly transformed modern connectivity, offering higher data rates, lower latency, and enhanced network reliability. Wireless Local Area Networks (WLANs) have become a cornerstone of this transformation and an integral part of 5G and Beyond (5G&B) wireless mobile networks [1]. These technologies are key components of next-generation Radio Access Networks (RAN), designed to meet the growing demand for 5G-grade enhanced Mobile Broadband (eMBB) services, massive Machine Type Communications (mMTC), and Ultra-Reliable Low-Latency Communications (URLLC) [2].

Under the 802.11 family of standards created and regularly updated by the Institute of Electrical and Electronics Engineers (IEEE), Wi-Fi continues to evolve in order to meet the exponentially growing user demands. Wi-Fi 5 (IEEE 802.11ac), released in 2014, faced challenges with real-time and mission-critical type of use cases, for addressing reliability and energy efficiency requirements especially in dense Internet of Thing (IoT) deployments [3,4]. The introduction of Wi-Fi 6 (IEEE 802.11ax) in 2021 marked a significant leap forward, offering data rates up to 10 Gbps, improved energy consumption, and enhanced reliability through Multi-User Orthogonal Frequency Division Multiple Access (MU-OFDMA), Multiple-Input Multiple-Output (MIMO), improved spatial reuse mechanisms, and higher-order modulation schemes [5]. Wi-Fi 7 (IEEE 802.11be), which was finalized in the end of 2024 with pre-standard products already in the market, promises to achieve data rates up to 46 Gbps, which is significantly higher than its Wi-Fi 6 predecessor, utilizing techniques such as Multi-Link Operation (MLO) for reduced latency making it ideal for real-time applications such as augmented/virtual reality (AR/VR) and remote surgeries [6–8].

Furthermore, next generation (NG) Wi-Fi technologies are expected to play an integral role in traffic offloading within the 5G&B RAN environment, particularly in indoor settings, where more than 80% of mobile data traffic is generated [2]. By seamlessly handling such a significant portion of data traffic, especially in high-density areas, NG Wi-Fi complements 5G&B networks, optimizing resource allocation and enhancing user experience [9]. With the increasing demands for high-speed connectivity in homes, offices, and public indoor spaces, traffic offloading via Wi-Fi can alleviate the burden on mobile network infrastructure, making it a critical component of the future integrated wireless ecosystem [1,2].

Despite the continuous advancements in Wi-Fi performance, security vulnerabilities remain a critical concern. Extensive research has consistently highlighted the strengths, weaknesses, and vulnerabilities inherent in Wi-Fi networks. To address these concerns, several security protocols have been developed, starting by Wired Equivalent Privacy (WEP), followed by Wi-Fi Protected Access (WPA), and its subsequent versions WPA2 and WPA3, with each providing varying levels of authentication and data protection [10,11]. While WPA2 remains the most widely deployed protocol, WPA3, despite being relatively new, represents the most secure option currently available [12].

Numerous attacks targeting Wi-Fi networks have been identified [11–13], with significant vulnerabilities found in WPA-TKIP (Temporal Key Integrity Protocol), including plaintext recovery attack [14], flawed random number generation attack [15], predictable password attack [16], offline attack targeting 4-way handshakes [17], man-in-the-middle attack [18], and downgrade attack [19]. A major concern is downgrade attacks, through which adversaries force networks to revert to weaker encryption standards, such as from WPA3 to WPA2, leaving them susceptible to known security flaws. With the fast-going rollout of Wi-Fi 6 and 5G networks, there is an urgent need to bolster security, particularly for WPA3 as the latest encryption standard, to effectively prevent unauthorized access as well as data breaches. The ability of address these security challenges is essential to maintain the reliability and integrity of NG Wi-Fi networks.

The discovery of the Key Reinstallation Attack (KRACK) vulnerability in the 4-way handshake [20] led to the development of WPA3 [21], which utilizes the dragonfly handshake, formaly defined as Simultaneous Authentication of Equals (SAE). WPA3 adoption remains relatively low, with only 0.84% of Access Points (APs) utilizing it even it has been a compulsory for the implementations of Wi-Fi since 2020 [21,22]. Nevertheless, a significant drawback of WPA3 lies in its transition mode (WPA3-TM) [23], which allows backward compatibility with WPA2 clients. This compatibility introduces vulnerabilities to downgrade attacks, denial-of-service (DoS) attacks and deprivation attacks [23–26]. The diverse landscape of network devices, with varying levels of security updates, further complicates the transition to more secure protocols. Therefore, the security of modern and NG Wi-Fi standards remains a critical issue, necessitating robust intrusion detection systems (IDS), in view of existing detection methods, which often struggle with dynamic security scenarios and may generate false-positive alerts [24].

To address these challenges, this paper introduces a novel hybrid adaptive approach based on machine learning (ML) for classifying incoming traffic to enhance downgrade attack detection in WPA3-protected networks. The proposed approach, ML-based Feature Selection and Thresholding for Downgrade Attacks Detection (MFST-DAD), integrates adaptive feature selection with dynamic thresholding, offering a more robust detection mechanism in varying network traffic patterns and practical scenarios in comparison to traditional fixed-threshold techniques.

The proposed MFST-DAD framework introduces a distinctive and purpose-driven approach by combining entropy-based adaptive feature selection with Median Absolute Deviation (MAD)-based dynamic thresholding, specifically designed to detect WPA3 downgrade attacks. Unlike conventional IDS models that employ static signatures or generic anomaly detection, MFST-DAD dynamically adapts to real-time traffic behaviors and is particularly effective in WPA3-Transition Mode (WPA3-TM), a security-critical scenario that remains underexplored in existing literature. To the best of our knowledge, this is the first work to implement this dual-adaptive mechanism on a custom-crafted WPA3 downgrade dataset generated under real-world experimental conditions. Our system not only achieves superior accuracy but also demonstrates higher resilience to false positives compared to recent IDS models such as those in [24,27,28], thereby offering a tangible advancement over prior approaches in both methodology and application scope. The key contributions of this paper are as follows:

- Introduce the MFST-DAD approach utilizing ML-based techniques for dynamic feature selection and threshold adaptation based on analysis of incoming traffic;
- Demonstrate how our hybrid method is capable of improving the security of WPA3 by incorporating applied, statistical, and ML-based thresholding models;
- Create a dataset used for evaluating downgrade attacks on WPA3-SAE and WPA3-TM, supporting the training as well as the assessment of ML models for intrusion detection;
- Showcase the performance of our proposed MFST-DAD the detection accuracy and reducing false alarms, enabling the system to learn complex attack patterns and distinguish them from normal network behavior.

To this end, the remainder of this paper is organized as follows. Sect 2 provides a comprehensive literature review of relevant Wi-Fi security challenges and existing solutions. In Sect 3, the details of our proposed MFST-DAD approach and each of its three phases are explained. The experimental setup and analysis of the generated dataset are presented in Sect 4. Sect 5 concludes the paper with potential future work.

## 2 Literature review

Security continues to be a persistent concern in Wi-Fi systems since their inception, driven by the need to adapt to evolving threats [29]. A central debate revolves around whether WPA3 represents a fundamental security breakthrough or an incremental improvement over WPA2 [30], particularly given its backward compatibility with WPA2 devices [23]. Early security flaws in WPA, such as vulnerability to dictionary attacks [16], prompted further scrutiny. He et al. [31] identified DoS vulnerabilities in the four-way handshake, providing a modular validity proof for IEEE 802.11i. However, the four-way handshake is prone to downgrade attacks as revealed by [25]. Consequently, clients will be forced to revert to the less secure WPA-TKIP with RC4 over WPA2 [32].

The work in [29] highlighted WPA3’s enhancements, including protection against key reinstallation attacks (KRACK) [20] and the introduction of SAE, while acknowledging remaining vulnerabilities. Subsequently, Vanhoef et al. [23] revealed "Dragonblood" vulnerabilities in WPA3, encompassing timing, DoS, cache, and downgrade attacks. As a result, the Wi-Fi Alliance issued updated security guidelines [21]. Several studies have shown the feasibility of offline and active dictionary attacks on WPA3, particularly during transition modes [33,34]. Recent research has been focusing on DoS attacks against WPA3-SAE [25,26] and vulnerabilities in the Management Frame Protection (MFP) leading to de-authentication attacks [35]. The work in [36] also introduced a time-memory trade-off as a new type of attacks that reduces the computational cost of breaking SAE-PK passwords.

Intrusion detection plays a critical role in network defense by classifying traffic as normal or malicious. Although signature-based IDS are regarded effective against known attacks, they are limited by their inability to detect novel threats. Anomaly-based IDS address this limitation by identifying deviations from usual behavior [37]. An approach for WPA3, detecting various attacks, including downgrade attacks, is proposed based on IDS signature [24]. However, relying on static thresholds only can lead to false positives and poor adaptability to dynamic network conditions. Thankappan et al. [38] developed a signal-based IDS for man-in-the-middle attacks, achieving high accuracy but with significant detection delays.

The application of ML in IDS has gained significant attention. ML-based IDS capture traffic packets to predict and detect attack classes [39,40]. Verma et al. [41] used feature extraction and classification algorithms to achieve high true positive rates (TPR) for DoS attacks. Saini et al. [27] developed a real-time ML-based IDS for enterprise environments, achieving high accuracy in flood detection. However, their reliance on frame number averages may lead to false positives, and they do not address downgrade attacks. Bhutta et al. [28] proposed a lightweight real-time IDS using LightGBM, demonstrating high accuracy and low latency. Shone et al. [42] applied deep learning to the NSL-KDD dataset, achieving high accuracy. Datasets like AWID and AWID3 [43] have been used for anomaly-based IDS development, but they primarily focus on WPA2 vulnerabilities. Thus, the lack of datasets specifically addressing WPA3 downgrade attacks poses another significant challenge.

Recently, the work by Balogun et al. [44] presented strategic defense mechanisms for IoT-based smart homes, emphasizing adaptive intrusion responses in distributed networks. Similarly, Nozari et al. [45] leveraged advanced Wi-Fi analytics for privacy-preserving monitoring, showcasing the power of multi-feature traffic analysis. These efforts underline the growing use of intelligent systems for securing Wi-Fi-based communication. Furthermore, Chatzisofroniou and Kotzanikolaou [46] conducted a security analysis of Wi-Fi Easy Connect, reinforcing that even newly adopted standards remain vulnerable. However, none of these approach directly address downgrade attacks in WPA3 or propose real-time, entropy-based detection mechanisms. Our approach proposed in this paper fills this gap by developing and validating a dedicated ML-based framework tailored to WPA3 downgrade vulnerabilities.

In summary, the existing literature highlights the limitations of current Wi-Fi security solutions, particularly the lack of downgrade attack-labeled datasets for WPA3. This necessitates updated resources that accounts for the susceptibility of WPA3 towards downgrade attacks. This research emphasizes the importance of both analytical and practical approaches, underscoring the need for a thorough investigation of downgrade attacks using WPA3-specific datasets and practical testing.

## 3 Methodology

**Fig 1:**
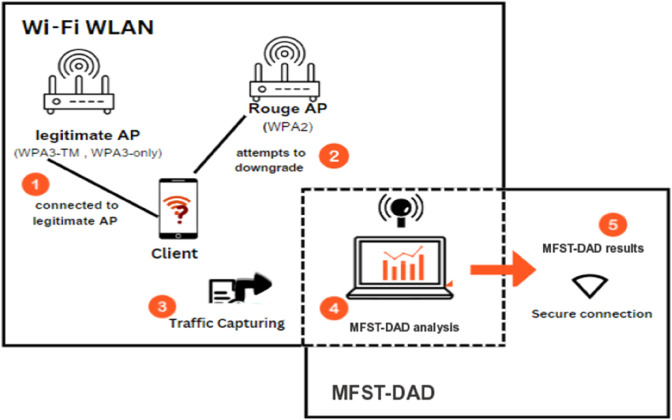
MFST-DAD system model.

The proposed ML-based Feature Selection and Thresholding for Downgrade Attacks Detection (MFST-DAD), illustrated in Fig 1, is designed to detect and counter downgrade attacks in WPA3. The MFST-DAD employs a hybrid approach that integrates statistical analysis, thresholding mechanisms, and ML-based adaptive feature selection to enhance WPA3 security. MFST-DAD combines network traffic capturing, thorough packet payload inspection, and real-time finding capabilities, which allows for improved classification of whether the activity is a downgrade attack or a normal one, with a concentration on adjustments according to the selected feature set. This set allows MFST-DAD to function across both WPA3-only and WPA3-Transition modes, ensuring comprehensive security.

**Fig 2:**
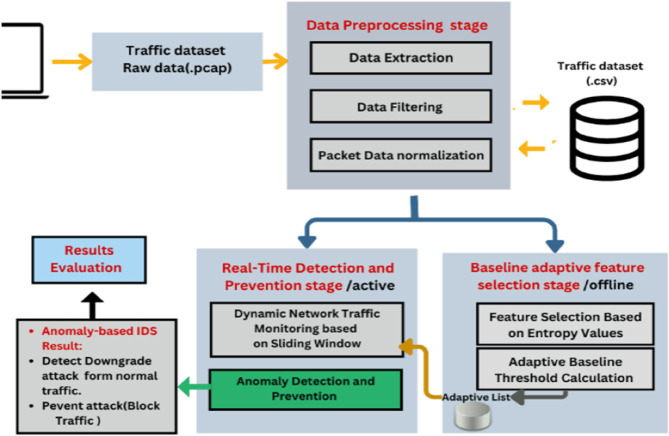
MFST-DAD design architecture.

The design architecture of the MFST-DAD approach, as depicted in Fig 2, comprises three distinct stages: (1) Traffic Data Preprocessing (TDP), (2) Baseline Adaptive Feature Selection (BAFS), and (3) Real-time Detection and Prevention (RDP). In the TDP stage, raw network packets are captured, and relevant packet details are derived and normalized to ensure data consistency. Subsequently, during the BAFS stage, the preprocessed dataset is analyzed to automatically choose features according to their entropy as well as a dynamic baseline threshold. This adaptive selection process allows the system to prioritize the most informative features for attack detection. Finally, the RDP stage employs ML models to classify incoming packets in real-time indicating either a downgrade attack or normal traffic, which consequently enables the system to take immediate preventative actions, and thus effectively mitigating the impact of detected attacks. We now proceed to elaborate on the specifics of each stage.

### 3.1 Assumptions and justifications

The MFST-DAD framework is designed with a set of foundational assumptions to ensure effective and reliable detection of WPA3 downgrade attacks, as follows:

- Downgrade attacks introduce measurable entropy deviations in key traffic features such as frame length, frame sequence, and Authentication Key Management (AKM) type, due to abnormal behavior caused by rogue access points.
- These entropy fluctuations serve as consistent anomaly indicators across both WPA3-SAE and WPA3-TM environments.
- The statistical profile of benign network traffic remains sufficiently stable over time, allowing adaptive thresholds based on MAD to effectively distinguish between normal and anomalous behavior.
- Real-time packet capture and feature extraction are feasible using standard monitoring tools in realistic wireless environments.

### 3.2 Traffic data preprocessing (TDP) stage

Traffic packets are recorded and used as an input dataset, whether they come from a healthy or compromised network. At the beginning, the network interface of the device is configured to monitor mode, which records every packet from the Wi-Fi connection in the Packet Capture (PCAP) file. Information including IP addresses, ports, headers, and payloads can be extracted from these packets. They are critical for developing a system to defend against downgrade assaults. Thus, frame properties are detected and chosen from the collected traffic information.

**Algorithm 1 Traffic data preprocessing (TDP).**

**Require:** Incoming Data (*I*_*T*_)

**Ensure:** Preprocessed Data (*P*_*T*_)

1: Extract data from *I*_*T*_, store in CSV file.

2: Classiy data based on specified parameters such as (frame type, source IP, ...etc.).

3: Normalize data using Eq (1)

4: // Utilize *P*_*T*_ in BAFS stage.

Algorithm 1 explains how traffic data is preprocessed. The incoming traffic (*I*_*T*_) is first analyzed, selected and scaled. Given that certain types of frames are of greater importance for any network discovery or IDS system, the classification step is performed based on frame type, for example, beacon frames which are a management frame sent by AP to discover the existence of a station. These frames carry useful information such as the SSID, MAC address, AKM type and count, etc., . Another important frame is the probe response which is a frame type that is sent in response to a probe request received from a client. Despite being ordered out one at a time, response frames contain the same AP abilities as do the beacons. Hence, we offer a more precise and sensitive view of the network’s behaviour by restricting and highlighting on these frames. This is done to make the values of the packets comparable and to ensure that all the features are given equal weight during the assessment. The packet feature values also have distinct scales and units. These differences in values may increase the outliers; for instance, the high feature numbers could surpass others features and result in a skewed discovery. This makes it possible to standardize the features to a normal range. We rely on the Z score given in Eq (1) [47] as a method of normalization during preprocessing.

$$Zs=\frac{X_{i}-\mu }{\sigma }$$ (1)

such that *X*_*i*_ represents the current feature value, *μ* is the mean value, *σ* is the standard deviation of feature *i*, and *Zs* is the normalized value for each feature. Once the Z-score normalization is finished, the distribution shape is maintained while ensuring the features are on a comparable scale.

### 3.3 Baseline adaptive feature selection (BAFS) stage

In the MFST-DAD approach, best adaptive features are selected as follows. First, the traffic dataset created in the previous TDP stage is downloaded and cleaned. The dataset files include the normal as well as malicious traffic data. Based on Algorithm 2, the preprocessed traffic data (*P*_*T*_) are used as input for the offline ML training and analysis. This stage involves several operations to identify features that enhance anomaly detection. Initially, the entropy of each feature vector is determined using Shannon’s entropy [48] as shown in Eq (2):

$$E=-\sum_{i}p_{i}\log_{2}p_{i}$$ (2)

such that *E* gives the feature vector’s entropy and *p*_*i*_ is the occurrence probability of each outcome, and the summation is taken over all the outcomes. the entropy pattern of features alters and is different from its typical values in the case of an attack. Such deviations provides the MFST-DAD approach with the required inference for understanding how an attack behaves and what is the type of impacted feature.

**Algorithm 2 Baseline adaptive feature selection (BAFS).**

**Require:** Preprocessed traffic data (*P*_*T*_) – for offline ML training

**Ensure:** Both the selected adaptive feature and the baseline threshold, stored in ($Adaptive_{set}$)

1: Evaluate the entropy for each feature vector *E*(*F*_*i*_) using Eq (2)

2: Evaluate the baseline threshold (*Th*_*i*_) for each feature vector *F*_*i*_.

3: **for** each $F_{i}\in P_{T}$ **do**

4:   **if** $E(F_{i})>Th_{i}$ **then**

5:    Combine *F*_*i*_ and *Th*_*i*_ to $Adaptive_{set}$

6:   **else**

7:    Drop the feature *F*_*i*_.

8:   **end if**

9: **end for**

10: // utilize the $Adaptive_{set}$ in RDP stage.

Consequently, the normal threshold is set to detect anomalous features by their entropy once calculating the entropy of all feature vectors. The normal traffic during normal operating conditions of the network is used as a reference model to develop the normal threshold. Any feature’s entropy that goes above this threshold is considered to be an anomaly and is believed to be related to downgrade attacks. The chosen anomaly features are stored in a set called $Adaptive_{set}$ which comprises a collection of features that are used to identify anomaly related to downgrade attack. Some problems are linked to the weaknesses of the static thresholding techniques when the environment is constantly changing especially in Wi-Fi network attacks. However, these static techniques are not efficient in handling the dynamics that are inherent in the process. In the attack behaviors, it may result in variables with high entropy and low entropy that cause false positives. To improve the accuracy and adaptability of anomaly detection, our method employs MAD for dynamic thresholding, which is robust to noise and outliers in traffic data. Specifically, for a given set of entropy values $X=\{X_{1},X_{2},\ldots ,X_{n}\}$, the MAD is computed as:

$$\text{MAD}=\text{median}(|X_{i}-\text{median}(X)|)$$ (3)

We then define an adaptive threshold *T* as:

T=median(X)+k×MAD (4)

where *k* is a tunable sensitivity parameter. A feature is flagged as anomalous if its entropy exceeds *T*. This adaptive mechanism allows the model to adjust dynamically to changes in traffic distribution, overcoming the limitations of fixed-threshold techniques, which often lead to poor performance in real-world, variable conditions such as WPA3-TM scenarios.

In the classification phase, traffic data is transformed into feature vectors $𝐱=[x_{1},x_{2},\ldots ,x_{n}]$, derived from the adaptively selected feature set. These vectors are input to a supervised classifier f(𝐱)→y∈{0,1}, where *y* = 0 indicates normal traffic and *y* = 1 denotes an attack. The Naive Bayes classifier, which demonstrated the highest performance in our evaluation, assumes conditional independence among features and applies Bayes’ Theorem:

$$P(y|𝐱)\propto P(y)\prod_{i=1}^{n}P(x_{i}|y)$$ (5)

Such probabilistic modeling enables efficient and interpretable decision-making, especially when working with high-dimensional network traffic data.

### 3.4 Real-time detection and prevention (RDP) stage

In this stage, the incoming traffic is monitored and analyzed in real-time and within a certain time window called the sliding window. Then, the data are correlated with the chosen $Adaptive_{set}$ from the BAFS stage and are used to improve the detection by including the characteristics most relevant to the current traffic. The window length of the sliding window is adjusted to fit the incoming data. To perform the detection, the threshold value is used to detect abnormal features. This threshold is dynamic with the ability to be adjusted according to the load to ensure the response is always suitable for the current condition. As soon as the detection threshold raises an alarm for a downgrade attack, then the packets are processed by classifier, which is beneficial when dealing with large bandwidth and various APs, where centralizing the tracking of all vulnerabilities is impractical. Hence, for the purpose of this paper, the data set obtained is used for ML training and validation. When features are less than the threshold, then the process is carried on to the next windows and appended to it, and so on until the end of the analysis. In addition, to counteract the attack, MFST-DAD acts on the MAC addresses that are involved in malicious activities and the weak authentication process. Thus, including the MAC address of the attacker in the blacklist prevents it from reestablishing the connection with the network. Accordingly, any further attempts made by a packet attack with the same MAC address are also denied, thus contributing to a preventive measure against threats and increasing the security of WPA3 networks.

### 3.5 Computational complexity analysis

To assess the theoretical efficiency of MFST-DAD, we provide a breakdown of the computational complexity for each component of the pipeline, as follows:

- **TDP Algorithm:** *O*(*n*), where *n* is the number of captured packets.
- **BAFS’s Entropy Calculation:** O(f·n), where *f* is the number of features, with each feature’s entropy is computed based on packet distributions.
- **BAFS’s MAD-based Thresholding:** O(f·logf), driven by the need to compute medians for threshold adaptation.
- **RDP’s ML Classification:** *O*(*m*), where *m* is the number of testing instances, assuming a Naive Bayes classifier.

The full MFST-DAD pipeline thus scales linearly with traffic size in both preprocessing and classification phases and remains efficient for real-time detection in medium-sized environments (e.g., under 1 million packets per session). While formal convergence guarantees are not applicable due to the non-iterative nature of the thresholding step, the system demonstrates stable performance and low latency in practice, as will be discussed in Sect 4.

## 4 Performance evaluation

For the evaluation of the performance of our proposed MFST-DAD approach, we established various experimentally configured scenarios. The purpose of these experiments was to create a comprehensive dataset that includes normal as well as attack-related network conditions. The generated dataset comprised packet captures from three distinct scenario types, enabling a thorough analysis of network behavior.

### 4.1 Experimental setup

**Fig 3:**
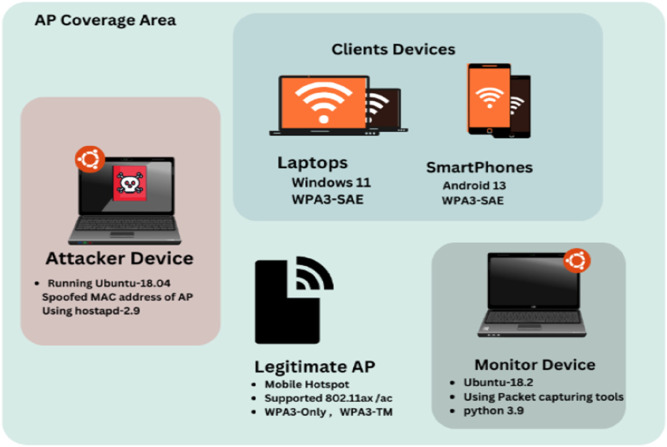
Experimental setup.

Fig 3 illustrates the experimental setup, which includes a mobile hotspot with the ability to switch between different WPA3 modes, connected client devices, a monitoring system, and an attacker node, with the latter two running Ubuntu. To generate the dataset, data packets from each scenario were labeled to identify specific attacks. The offline stage focused on analyzing input frames, particularly targeting downgrade attacks. The generated dataset contained packet records from the following attack scenarios: WPA3-SAE and WPA3-TM. In addition, the dataset included packets sent during the attacks and recorded by the sniffing device. To provide an extensive overview of network traffic during different circumstances, packets from a standard network scenario and transient interruption scenarios were included.

Currently, there is no publicly available dataset that captures WPA3-specific downgrade attacks, especially those involving WPA3-TM. To address this gap, we carefully designed and generated a custom dataset that reflects real-world WPA3 behavior under both normal and attack conditions. Our dataset includes the five different experimental scenarios explained below. These controlled scenarios were designed to ensure a realistic emulation of downgrade attack vectors and benign traffic patterns across multiple security settings. Although the dataset is limited in size, it was purpose-built to focus on key entropy-affecting features (e.g., frame types, AKM counts, sequence intervals) relevant to WPA3 security. This focused dataset enabled us to demonstrate the efficiency of our adaptive detection framework. As part of future work, we intend to integrate larger-scale datasets such as AWID3 and simulate WPA3 extensions using packet injection and augmentation techniques to further enhance the generalizability and robustness of the model.

The following describe the experimental conditions used to generate a dataset from both regular and attacked Wi-Fi WLAN connections. In data capture, the AP’s mode was also repeatedly reconfigured to emulate downgrade attack behaviours without actually performing any attacks.

- **Scenario 1**: Normal Operation
  - **Security Standard**: WPA3-SAE
  - **Attack Type**: None
- **Scenario 2:** Normal Operation
  - **Security Standard**: WPA3-TM
  - **Attack Type**: None
- **Scenario 3:** Attacked Condition
  - **Security Standard**: WPA3-SAE, WPA2
  - **Attack Type**: Downgrade
- **Scenario 4:** Attacked Condition
  - **Security Standard**: WPA3-TM, WPA2
  - **Attack Type**: Downgrade
- **Scenario 5:** Temporary Disruption: To reduce false positives
  - **Security Standard**: WPA3-TM and WPA3-SAE
  - **Attack Type**: Normal

### 4.2 Data representation and analysis

The analysis focuses on examining frames from various Wi-Fi scenarios as follows:

- **Type-0:** refers management frames
- **Type-1:** refers to control frames
- **Type-2:** refers data frames

**Fig 4:**
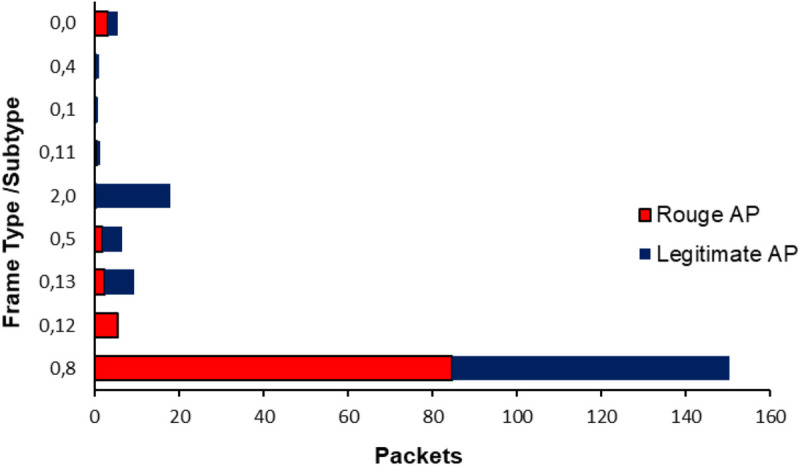
Analysis of frame types and their frequency of occurrence.

Analyzing these frames along with their subtypes helps to avoid incorrect Wi-Fi traces by verifying the frame type distribution. Fig 4 illustrates that the dataset comprises 24 features, including frame statistics from rogue APs and legitimate APs. For rogue APs, beacon which is subtype 8 of management frames are the most common frames, constituting approximately 90% of the total frames, then the deauthentication frames which is subtype 12 of management frames with 5%, and only 0.06% are EAPOL frames which is subtype 0 of data frames. In contrast, the frame distribution for a legitimate AP is different, with beacons still being the most frequent at about 50%. This distribution is typical for network traffic, as beacons announce the AP’s availability. There is also a significant presence of EAPOL packets at around 15%. EAPOL frames are generally used for establishing and managing secure associations, and their reduced occurrence during attacks indicates a timeout or failure in the connection setup process.

**Fig 5:**
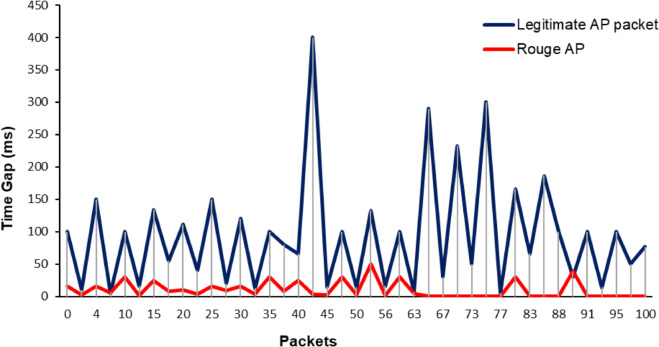
Analysis of time gaps between Wi-Fi packets.

Fig 5 illustrates the time gaps between Wi-Fi packets, highlighting the unique behavior of packets in comparison between legitimate and rogue APs. Accordingly, it is recommended to make a consistency check on the recorded traffic to identify any malicious activity, such as quick advertising by a rogue AP and minimal advertising by a legitimate AP. This discrepancy arises because a legitimate AP, unlike a rogue AP, spends less time advertising itself and more time on connection setup and data packet transmission, resulting in fewer frames.

The dataset used in this study was generated through controlled experiments that simulated downgrade attacks in WPA3-secured networks, incorporating both WPA3-SAE and WPA3-TM. The traffic was captured using real wireless devices configured in both normal and adversarial conditions. Each session was carefully labeled as either "normal" or "attack" based on the state of the network and the behavior observed during the session. To ensure diversity and realism, we varied the client devices, access point configurations, and the timing and nature of attacks. This allowed us to capture a wide spectrum of traffic behavior, including frame headers, packet types, authentication protocols (e.g., SAE, WPA2-PSK), and time-based anomalies.

The dataset consists of three distinct traffic scenarios:

- **WPA3-SAE Dataset:** Contains 2,000 frames and 16 features, simulating both normal traffic and downgrade attack conditions.
- **WPA3-TM Dataset:** Contains 2,313 frames and 16 features, simulating downgrade attacks and standard WPA3-TM transitions.
- **Temporary Disruption Dataset:** Contains 266 frames as samples and 16 features, representing normal but transient disruptions (e.g., brief AP changes or packet loss).

All data was collected from real hardware under repeatable conditions and includes session-level variability, making the dataset both diverse and representative of practical WPA3 deployment scenarios.

### 4.3 Results and discussion

#### 4.3.1 Results analysis.

**Fig 6:**
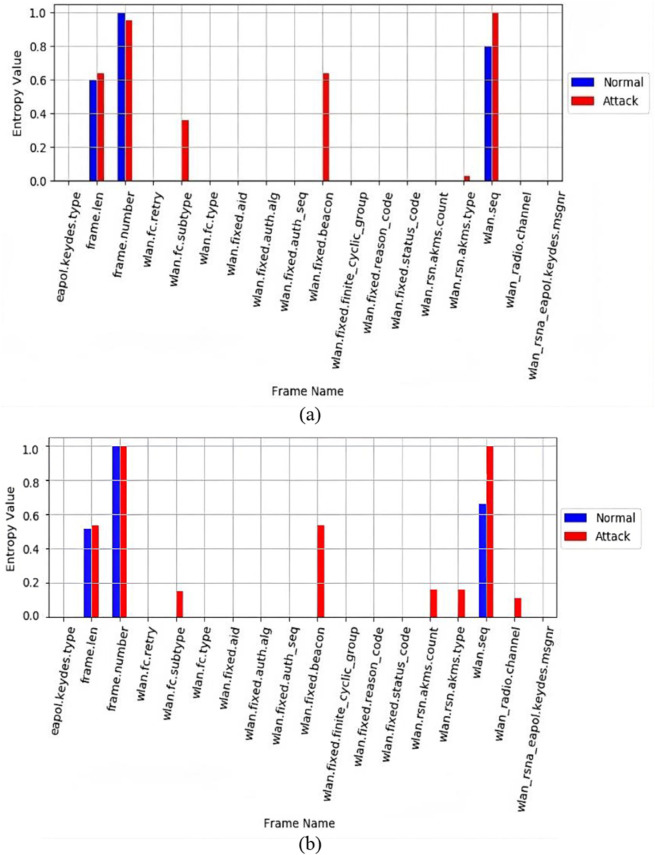
Entropy results analysis of MFST-DAD with (a) WPA3-SAE and (b) WPA3-TM.

To evaluate the effectiveness and accuracy of our proposed MFST-DAD approach, we conducted a series of experiments, and the outcomes are thoroughly analyzed in this subsection. The efficacy of feature selection is determined through a comparative analysis of entropy statistics. Additionally, we examine the real-time detection and prevention capabilities of our system and evaluate the performance of the ML-classifier. Notably, our BAFS algorithm demonstrates strong performance in conjunction with entropy statistics. As depicted in Fig 6(a) and 6(b), we present the entropy measurements for two APs: one operating exclusively in WPA3-SAE mode and the other in WPA3-TM mode. In both scenarios, we analyze a honeypot AP in WPA3-SAE mode under normal operating conditions, juxtaposed with the same legitimate AP under attack by a rogue AP utilizing WPA2. Under normal conditions in WPA3-SAE mode, the feature entropy exhibits lower values, indicating a stable feature vector within a secure network. This consistency in entropy during usual operation contrasts sharply with the variability shown in the attacked scenario.

In both the attacked and normal scenarios, modifications are visible in AKM types; frame length, frame sequence, and beacon intervals. Under the attacked scenario, the entropy of AKM types describes malicious attempts using various authentication algorithms whilst supporting WPA2-PSK. The attacker also affected frame length and sequence variations, indicating disturbances in normal communication patterns. Furthermore, changes in beacon intervals show disturbances in typical broadcasting behavior. During the normal scenario, the AP emits a beacon that contains the information about the supported AKM types, which are WPA3-SAE and WPA2-PSK authentication, and the AKM count is equal to two. The entropy examination reveals typical patterns of secure behavior, with consistent AKM type, AKM count, beacon interval entropy, and frame sequence entropy.

**Fig 7:**
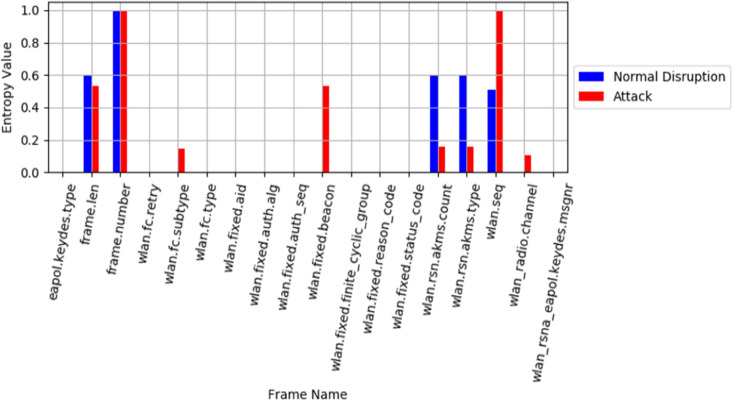
Entropy results analysis of MFST-DAD via temporary condition.

In contrast, when considering the results of the temporary disruption scenario illustrated in Fig 7, in which the AP briefly switched its security parameters from WPA3-TM to WPA3-SAFE. This change resulted in a significant shift in the AKM count and entropy types, similar to the attack scenario. Nevertheless, entropy values and feature sets varied. Interestingly, despite this momentary disruption, no changes were noticed in the frame sequence, intervals, or length, unlike the attack behaviors. This distinction helps differentiate between attack behaviors and transient conditions, thereby reducing false negatives.

To assess the robustness and adaptability of MFST-DAD in real-world, unstable environments, we simulated two common network disturbances: controlled packet loss and transient AP switching. Packet loss was introduced at varying levels up to 15%, while transient AP changes were used to emulate handoff scenarios or brief disconnections. MFST-DAD maintained a high detection accuracy of 97.6% under these perturbed conditions, with only a 1.4% increase in false positives compared to the baseline. Thus, there is minimal degradation in the model’s resilience and adaptability, particularly in the context of WPA3-TM traffic, which is more susceptible to such transient anomalies, which confirm that MFST-DAD can generalize beyond ideal conditions and remain effective under typical wireless network variability.

Additionally, and too ensure a robust and unbiased evaluation of MFST-DAD, we adopted a two-stage hold-out validation approach. After preprocessing, the dataset was split into two operational phases to mimic real-time system behavior and offline training/testing.

In the first stage, a subset of the data, comprising both normal and attack traffic, was reserved to establish baseline behavior for entropy and threshold modeling. The remaining portion was treated as live traffic and analyzed incrementally using a sliding window mechanism, which emulates real-time packet flow and detection response.

In the second stage, we created an integrated dataset combining all labeled traffic (normal and attack) and applied a standard hold-out validation procedure. Specifically, 70% of the data was randomly allocated for training, while the remaining 30% was used as an independent test set. This split ensured that no overlap occurred between training and testing, thereby minimizing the risk of overfitting or data leakage.

Next, the previously described adaptive thresholding technique was applied. For each feature vector, a threshold value was set to identify anomalies. Anomalous features were selected and added to an adaptive set. If a feature’s entropy exceeded the threshold, it was flagged as an anomaly. The approach then evaluated a traffic dataset by mapping it to the adaptive feature set, learning from the dynamic traffic. When the window size was set to the total traffic length, it calculated feature entropy and set the anomaly detection threshold.

**Fig 8:**
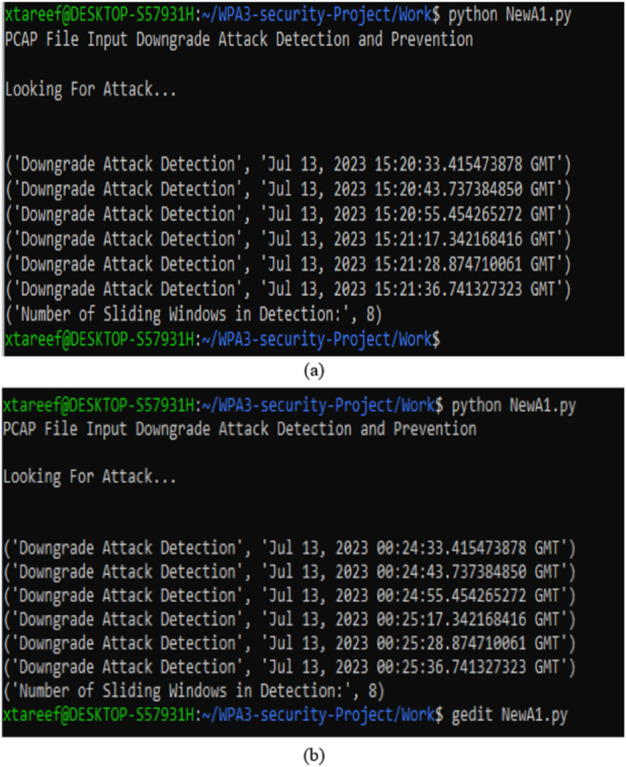
Response of MFST-DAD’s RDP algorithm to data captured from (a) WPA3-SAE only and (b) WPA3-TM attack scenarios.

**Fig 9:**
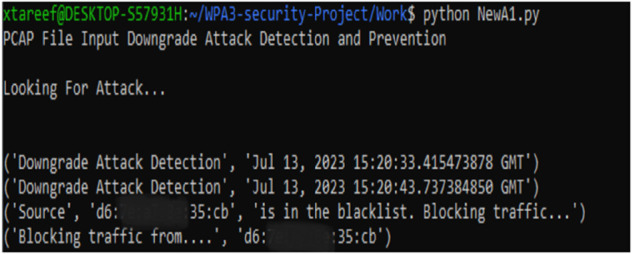
Real-time prevention response of MFST-DAD with adaptive blacklist mechanism.

As shown in Fig 8(a) and 8(b), the proposed MFST-DAD approach effectively identifies downgrade attacks using its RDP algorithm in both WPA3-SAE and WPA3-TM, providing precise detection times. Additionally, MFST-DAD prevents downgrade attacks through an adaptive blacklist mechanism, which immediately bars any identified threat from accessing the network. This prevention measure proved effective across all simulated scenarios, as shown in Fig 9. The blacklist also functions in a virtual environment on the same platform, demonstrating its feasibility without requiring manufacturer-specific solutions.

**Fig 10:**
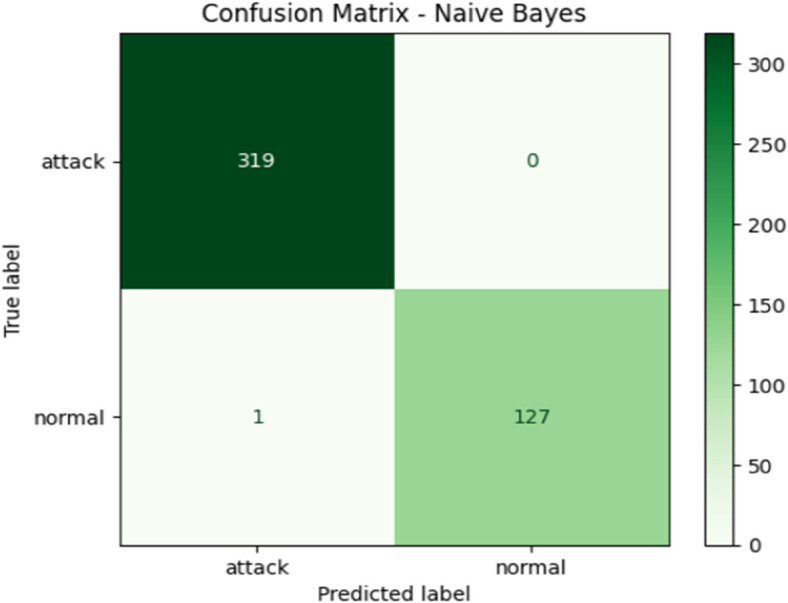
Confusion matrix of the Naive Bayes classifier used in MFST-DAD.

To further enhance attack prediction accuracy, we integrated ML-based classifiers into MFST-DAD’s thresholding stage. Table 1 compares the performance of Naive Bayes (NB), K-Nearest Neighbors (KNN), Support Vector Machine (SVM), and Stochastic Gradient Descent (SGD). In comparison, we see that NB exhibited superior performance across accuracy, F1-score, recall, and precision, indicating its strong capability in identifying network attacks within the framework of our proposed MFST-DAD approach. Additionally, Fig 10 illustrates the confusion matrix, where 30% of samples were used for testing and 70% for training. The results demonstrate high classification accuracy, with the model correctly identifying 319 out of 319 attack instances and 127 out of 128 normal instances. Only a single normal instance was misclassified as an attack, while no attack instances were misclassified as normal. This reflects the strong performance of the proposed MFST-DAD approach in both precision and recall, highlighting its effectiveness in distinguishing between attack and normal traffic within the proposed detection system.

**Table 1:** Evaluation results of ML-based classifier within MFST-DAD framework.

Classifier	Accuracy (%)	Recall (%)	Precision (%)	F1 score (%)
NB	99.8	97.7	99.7	99.8
KNN	97.9	97.7	97.4	97.5
SVM	85.9	78.3	86.9	81
SGD	78.1	84.5	78.5	77.2

In addition to traditional ML classifiers (NB, KNN, SVM, SGD) discussed in Table 1, we assess the relative effectiveness of the proposed MFST-DAD framework by comparing its performance against several established intrusion detection techniques, as shown in Table 2, we notice that the detection rate and accuracy have been enhanced in comparison. There are also some disadvantages of the signature based IDS proposed by Dalal et al. [24] and Saini et al. [27] that can detect abnormal packets and are accurate. Bhutta et al. [28] also achieved high accuracy with a lightweight Wi-Fi IDS with LightGBM, including downgrade attack detection. Another recent approach, SAECRED [49], which uses commercial off-the-shelf (COTS) Wi-Fi access points to detect downgrade attacks based on beacon spoofing and signal anomalies. Although their method is deployable without custom datasets or training, it does not employ supervised learning, instead it applies context-sensitive fuzzing to discover downgrade and parsing bugs in WPA3 SAE implementations. While SAECRED demonstrates high protocol coverage and uncovers critical flaws, it does not offer accuracy metrics or classifier-based detection performance.

**Table 2:** Benchmarking with state-of-the-art related works.

Work	Approach	Accuracy	Disadvantage	Scope
Dalal et al. [24]	Signature-based IDS, attack vector	>99%	Possible false positives during AP restart or reconfiguration	Abnormal packet detection, Downgrade attack
Saini et al. [27]	Signature-based IDS, Random Forest based ML-classifier	>99%	Spike in the average number of packets may results in false positives Focus only on flood attacks	Flood attacks
Bhutta et al. [28]	Lightweight Wi-Fi IDS	99.7%	Limited to Downgrade attack	Wi-Fi attack, WPA3 flood attacks
Proposed MFST-DAD	Adaptive feature set and dynamic thresholding	99.8%	Focus only on downgrade attack	Downgrade attack in WPA3-SAE and WPA3-TM

In contrast, our proposed MFST-DAD achieved a detection accuracy of 99.8% in real-world WPA3 downgrade attack in both WPA3-SAE and WPA3-TM scenarios, demonstrating its high precision and robustness in conditions that are overlooked by most existing approaches, and illustrating its advantages in adaptive feature handling and attack-specific accuracy. However, there are some limitations of the approach proposed in this paper, including the scope of the approach; nevertheless, it has a significant improvement in addressing Wi-Fi vulnerabilities if compared to other approaches.

#### 4.3.2 Statistical significance and confidence intervals.

Further, to assess the robustness of our classification results, we conducted statistical significance testing using McNemar’s test to compare the performance of MFST-DAD against baseline classifiers, including KNN and SVM. The test evaluates whether the difference in classification outcomes between two models is statistically significant. When comparing MFST-DAD (NB classifier) against KNN and SVM, McNemar’s test produced a p-value < 0.01 in both cases, indicating that the performance improvements of MFST-DAD are statistically significant at the 99% confidence level. Additionally, we computed 95% confidence intervals (CI) for all key metrics. For NB, the model that achieved the best results, the 95% CI for classification accuracy was [99.5%, 100%], supporting the robustness of the results.

#### 4.3.3 Ablation study.

To evaluate the individual contributions of key components in the MFST-DAD framework, we conducted a series of ablation experiments by selectively disabling one module at a time and observing the resulting performance impact. The results are summarized below:

- **Without entropy-based feature selection:** Accuracy dropped significantly to 92.1%, indicating that adaptive feature selection is essential for eliminating irrelevant or noisy features and improving classifier performance.
- **With static thresholding instead of MAD-based dynamic thresholding:** The system exhibited a 17% increase in false positive rate, demonstrating the importance of adaptive thresholding in handling traffic variability.
- **Without MAC-based blacklist prevention:** Attack recurrence increased by 28%, which shows the important role of the prevention mechanism in reinforcing long-term network resilience.

These findings confirm that each component involved in the design of our proposed MFST-DAD approach, including entropy-based selection, MAD-based thresholding, and MAC-based prevention, contributes meaningfully to its robustness, precision, and practicality. The combined approach outperforms simplified versions in both detection accuracy and defense continuity.

## 5 Conclusions and future work

The paper presented a novel hybrid approach designed to detect and prevent security vulnerabilities arising from the backward compatibility of WPA3 in modern and next generation (NG) Wi-Fi networks, which is a challenge not adequately addressed in existing literature. Our approach integrates statistical analysis, adaptive thresholding, and machine learning (ML) to classify downgrade attacks and distinguish them from normal network behavior. By incorporating entropy statistics and MAD-based thresholding, it effectively identifies critical features and enhances accuracy through ML-based classification. Experimental results demonstrated the reactivity and accuracy of the proposed approach, highlighting a robust intrusion detection capabilities. Specifically, the integration of the Naive Bayes classifier within our hybrid approach achieved an impressive accuracy of 99.8% in detecting downgrade attacks across both WPA3-SAE and WPA3-TM modes.

While the MFST-DAD system demonstrated high accuracy in controlled experimental setups, several limitations remain. First, the dataset was generated in a lab environment and may not fully reflect the heterogeneity of real-world WPA3 deployments, especially in enterprise-scale networks. Second, although the system adapts to typical downgrade behaviors, sophisticated adversaries using spoofed MAC addresses or low-and-slow tactics may bypass detection. Lastly, generalization to other network types (e.g., WPA3-Enterprise in mesh networks) has not yet been tested and is left for future exploration.
